# Effects of FamilyDoctor Concept and Doctor-Patient Interaction Satisfaction on Glycaemic Control among Type 2 Diabetes Mellitus Patients in the Northeast Region of Peninsular Malaysia

**DOI:** 10.3390/ijerph17051765

**Published:** 2020-03-09

**Authors:** Noorfariza Nordin, Suhaily Mohd Hairon, Najib Majdi Yaacob, Anees Abdul Hamid, Norzaihan Hassan

**Affiliations:** 1 Department of Community Medicine, School of Medical Sciences, Universiti Sains Malaysia, Kelantan 16150, Malaysia; drnoorfariza@student.usm.my; 2 Unit of Biostatistics and Research Methodology, School of Medical Sciences, Universiti Sains Malaysia, Kelantan 16150, Malaysia; najibmy@usm.my; 3 Primer Health Unit, Kelantan State Health Department, Kelantan 15590, Malaysia; dranees@moh.gov.my; 4 Kota Bharu Health Clinic, Kota Bharu District Health Office, Kelantan State Health Department, Kelantan 15586, Malaysia; norzaihanhassan@yahoo.com.my

**Keywords:** family doctor concept, primary healthcare, doctor-patient interaction, patient satisfaction, glycaemic control, Type 2 Diabetes Mellitus

## Abstract

The implementation of Family Doctor Concept (FDC) to restructure the primary healthcare systems in Malaysia were expected to enhance patient’s satisfaction on doctor-patient interaction and subsequently improved glycaemic control among Type 2 Diabetes Mellitus (T2DM) patients. Thus, this study aims to determine the difference in doctor-patient interaction satisfaction between T2DM patients attended FDC-implemented clinic vs non-FDC clinics, and to determine the association between FDC-implemented clinic and doctor-patient interaction satisfaction towards glycaemic control. A cross-sectional study was conducted throughout 10 districts in Kelantan from February until May 2019 using interview-guided *Skala Kepuasan Interaksi Perubatan-11* (SKIP-11) and proforma checklist. Data were analyzed using SPSS ver.24. Chi-square statistic used to determine the difference in doctor-patient interaction satisfaction between both clinics type. Multiple logistic regression used to examine the association between FDC-implemented clinic and doctor-patient interaction satisfaction towards glycaemic control. Twenty primary health clinics involved, and 772 T2DM patients recruited. FDC clinics attendees has higher proportion of satisfaction (40.1%) compared to non-FDC attendees (33.7%) (*p* = 0.070). Multiple logistic regression confirmed the association of FDC-implemented health clinics (Adj. OR 1.63, *p* = 0.021), and doctor-patients interaction satisfaction (Adj. OR 1.77, *p* = 0.005) towards glycaemic control. Hence, strengthening of FDC in primary healthcare and improve the doctor-patient interaction satisfaction were essential to escalate good glycaemic control.

## 1. Introduction

Type 2 Diabetes Mellitus (T2DM) is a known non-communicable disease which is prevalent in Malaysia [1]. This metabolic disorder causes major health issues, together with social and economic impacts. The Malaysian National Health & Morbidity Survey (NHMS) reported the prevalence of diabetes increased from 14.9% in 2006 to 17.5% in 2015, respectively [1,2], and it was projected to rise to 21.6% by the year 2020 [3]. The aim of managing T2DM is to achieve good glycaemic control measured by glycated haemoglobin (HbA1c) level within or close the normal range [4]. Generally, good glycaemic control is classified as HbA1C of ≤6.5% [5].

Malaysia initiated the Family Doctor Concept (FDC) in 2013 to provide comprehensive services and strengthen its primary healthcare system [6]. The FDC offers personalized care, which subsequently able to improve the T2DM patient’s outcome following a good doctor-patient relationship where diagnosis, complications, and follow-up can be addressed properly [6,7]. Thus, Kelantan State Health Department implemented the FDC concept starting 2015, by which a total of 33 from 85 government primary health clinics in Kelantan has been gazette as FDC-implemented clinics in 2018, and the remaining health clinics (non-FDC clinics) are still functioning as per current practice. It is a restructuring of primary health services; its infrastructure and equipment, healthcare personnel, clinic’s floor set-up, clinic’s physical space and scheduled appointment; to ensure patients and population are taken care by specific Primary Healthcare Team (PHCT) according to “zone” [6]. The population under the clinic’s operational area were group into smaller and specific cluster named “zones” which demarcated by specific streets or river [6]. Each zone consists of 3000 to 15,000 population and assigned to specific PHCT team under FDC-implemented clinics. In average, the team per zone comprised of 1–3 medical officers, 2–4 nurses, 1–3 community health nurses, 1–2 medical assistant [6]. Preferably, the appointed nurses and/or medical assistant are trained as a diabetic educator. The number of PHCT varies according to the number of zones for each primary health clinics. Meanwhile, speciality services such as the Family Medicine Specialist (FMS), pharmacist, dietician, physiotherapist therapist, occupational therapist, radiology and laboratory are being shared across all zones. Personalized care is a term used when patients were seen by the same set of PHCT each time they seek treatment at the FDC-implemented clinics.

As for non-FDC clinics, it follows the current primary healthcare practice, which has no dedicated team and no specific geographical zoning. The current practice requires patients with chronic illness, antenatal and other acute diseases to be managed separately, and no personalized care involved. There were no resident Family Medicine Specialist, fewer numbers of medical officers and limited diabetic educators. Other specialities were also shared across the district. Nonetheless, the availability of equipment such as fundus camera and x-ray equipment was severely limited at the non-FDC clinics. Patients need to get an appointment and mobilize to the nearest FDC clinics in the district to undergone fundus camera or x-ray examination when needed, and the results will be analysed by their non-FDC doctors later. In addition, patients with poor glycaemic control who being treated at non-FDC clinics will be given an appointment to be seen by the visiting FMS and other specialities when needed as decided by the medical officer.

Following the implementation of FDC, the consultation time are expected to be reduced as patients are being managed by the same doctor who already known and understand the patients’ social issues such as their living condition [6]. This will subsequently increase patient satisfaction on doctor-patient interaction which can further increase adherence and improved understanding towards diabetic management, hence improved diabetic control [8,9,10]. Patients satisfaction on doctor-patient interaction is about the patient’s feeling regarding their treating doctor and the patient’s experience compared to their expectations [9]. The evaluation of the satisfaction of doctor-patient interaction is needed to guide organizational strategies in achieving a better diabetic outcome [11]. One of the tools to assess the satisfaction of doctor-patient interaction from the patient’s perspective is the Medical Interview Satisfaction Scale (MISS-21) questionnaire. This questionnaire has been translated and validated to Malay language, hence valid assessment of doctor-patient interaction satisfaction among Malaysian population can be made.

There are several significant factors associated with glycaemic control which has been revealed by many studies such as age, sex, duration of diabetes and educational level [4,12,13,14,15,16]. However, to the best of our knowledge, there is scarce local evidence that examines the relationship between FDC implementation and doctor-patient interaction satisfaction towards glycaemic control among T2DM. Hence, this study aims to determine the difference in doctor-patient interaction satisfaction between T2DM patients attended FDC-implemented clinic vs non-FDC clinics, and to determine the association between FDC-implemented clinic and doctor-patient interaction satisfaction towards glycaemic control among T2DM in Kelantan population.

## 2. Materials and Methods

### 2.1. Study Design and Study Location

This was a cross-sectional study that has been conducted from February until May 2019 using proforma and the Malay version MISS-21 questionnaire named *Skala Kepuasan Interaksi Perubatan-11* (SKIP-11). This study was conducted throughout all 10 districts in Kelantan involving two primary health clinics for each district, an FDC-implemented health clinic and a non-FDC health clinic.

### 2.2. Study Participant

The inclusion criteria for this study were; T2DM patients aged 18 years old and above, have been diagnosed and follow-up at selected health clinics before the year 2017, received follow-up care at selected health clinics at least twice within the last one year, and able to understand the Malay language. Pregnant women with pre-existing T2DM were excluded from the study because the management for antenatal was more comprehensive.

### 2.3. Sampling Method

T2DM patients were the sampling unit for this study. Firstly, simple random sampling performed to select one FDC-implemented health clinic and one non-FDC health clinic from each district in Kelantan. Subsequently, systematic random sampling was conducted on every data collection day to select the participants. One out the first two patients who presented to the participating clinics were randomly selected from the clinic’s registration counter and subsequent participant selected at regular two-unit intervals.

### 2.4. Data Collection

Data was collected by using a proforma checklist and Medical Interview Satisfaction Scale-21 [*Skala Kepuasan Interaksi Perubatan-11* (SKIP-11)] questionnaire. Proforma checklist was used to collect information on the sociodemography (age, sex, race, marital status, education level, employment status), diabetic profile (duration of diabetes, latest HbA1c reading, number of diabetic complications), and clinic’s characteristics (duration of FDC’s implementation, number of diabetic patients attended clinic per day, total number of clinical staff, availability of fundus camera and x-ray machine). The information regarding sociodemographic and diabetic profile were obtained from the individual patient’s diabetic records. Meanwhile, information regarding clinic’s characteristics was obtained from the Primer Health Unit, Kelantan State Health Department.

The SKIP-11 questionnaire was used to measure the satisfaction on doctor-patient interaction. It was originated from MISS-21 in the English language and has been translated to Malay version and validated by Abd Aziz et al. with Cronbach alpha of 0.669 [11]. Data were collected through interview guided. This questionnaire consists of 11 items in three subdomains, namely distress relief, rapport and interaction outcome. For each of the subdomains, distress relief was constructed by four items that measure information provision by the doctor, rapport constructed by four items which measure patient’s confidence in the doctor, and interaction outcome constructed by three items that reflect the doctor’s communication skills and adherence intent [9,11]. Each item scored on a 5-point Likert scale which for positively worded items, score 1 for ‘strongly disagree’ and score of 5 for ‘strongly agree’. The score was reversed for negatively worded items. For the overall SKIP-11 score, the minimum total score was 11, and the maximum score was 55. For the subdomain, distress relief and rapport have minimum score of 4 and maximum of 20 each; interaction outcome has minimum score of 3 and maximum of 15. The score was then categorized into satisfied and unsatisfied based on overall items and items for each subdomain. The total SKIP-11 score (overall item) were categorized into satisfied if the scores was more than 44. For the subdomain, distress relief score more than 16, rapport score more than 16, and interaction outcome score more than 12 were categorized as satisfied [9]. The dependent variable was glycaemic control which categorized as good if the HbA1c reading was ≤6.5%.

### 2.5. Operational Definition

Glycaemic control was defined using the latest HbA1c (within the past 6 months of enrolment) recorded in the individual patient’s diabetic record and classified into good control when HbA1c was ≤6.5%; poor control when reading >6.5% [5]. FDC-implemented clinics were primary health clinics gazetted by the Kelantan State Health Department to implement the Family-Doctor Concept for the management of patients with diabetes from the year 2015 till 2017.

### 2.6. Statistical Analysis

Data entered and analyzed using SPSS ver. 24 (IBM Corp. Released 2016. IBM SPSS Statistics for Windows, Version 24.0. Armonk, NY, USA: IBM Corp.). Exploratory data analysis was performed to check for missing values and distribution of numerical data. Numerical data was said to be normally distributed when the histogram with overlaid normal curve showed an approximately normal bell-shaped curve, absence of extreme outliers on boxplot, and a non-significant test of normality (Kolmogorov-Smirnov and Shapiro-Wilk test). Data were presented as; mean (SD) for numerical variables, and, number (*n*) and percentage (%) for categorical variables. Chi-square statistic used to determine the difference in doctor-patient interaction satisfaction between T2DM patients attended FDC-implemented clinic vs non-FDC clinics. The proportion in satisfaction category, overall SKIP-11 and its subdomains were described in column percentage.

Simple logistic regression used to identify important factors associated with good glycaemic control at the univariable level. The factors include age, sex, race, marital status, employment status, duration of diabetes, and average T2DM attended clinics per day. The aim of conducting this univariable analysis is to build a parsimonious logistic regression model. Variables with a *p*-Value of less than 0.25 were then included in confirmatory models to test the association between FDC clinics and doctor-patient interaction satisfaction with glycaemic control. Two confirmatory models were tested using the enter method of multiple logistic regression while adjusting for other factors. Fitness of the models was tested using Hosmer Lemeshow goodness of fit test with *p*-Value > 0.05 indicated that the model was fit. The classification table and receiver operating characteristic (ROC) curve were also used to determine the fitness of the models. Classification table with an overall correctly classified percentage of 80.0% and above; and ROC curve with area under the curve of 0.7 and above indicated that the models were fit. All possible two-way interaction between variables included in each model was examined. The final models were presented as an adjusted odds ratio (OR), 95% confidence interval (CI) and *p*-Value. A two-tailed *p*-Value of less than 0.05 was considered statistically significant.

### 2.7. Ethical Approval and Consent to Participate

Ethical clearance was obtained from the Human Research Ethics Committee of the University Sains Malaysia USM/JEPeM/18100579 and the Medical Review and Ethical Committee from the National Institute of Health, Ministry of Health NMRR-18-2895-44351 (IIR). Written approval obtained from the Kelantan State Health Department. The study objectives were explained by the interviewer before obtaining the participant’s written informed consent. All participant was informed that they would be interviewed for 15–20 min, and they can terminate their participation at any moment. The result from this study was not be informed personally to the participant but shall be published into the journal. All participant was given a token of appreciation for their time to be interviewed.

## 3. Results

A total of 772 T2DM patients from 20 selected primary health clinics involved in this study. Majority of them were female, Malay, married and unemployed. Patient’s profile was shown in Table 1. All the variables show no significant difference except for race. Meanwhile, the profile of the clinics was shown in Table 2. The number of healthcare personnel, including the medical officers, pharmacist and physiotherapists were higher in FDC clinics, and it differs significantly between FDC and non-FDC clinics (*p* = 0.005, *p* = 0.001 and *p* < 0.001 respectively). FDC clinics have a significantly higher average of T2DM patients visited clinic per day as compared to non-FDC clinics (*p* = 0.018). Meanwhile, the majority of the FDC clinics were equipped with a fundus camera and an x-ray machine at site.

### 3.1. Doctor-Patient Interaction Satisfaction among T2DM Patients

In general, the doctor-patient interaction satisfaction was low among T2DM patients who attended primary health clinics in Kelantan as only 288 (37.3%) satisfied. However, patients who attended FDC clinics have a higher proportion of satisfaction (40.1%) as compared to patients who attended non-FDC clinics (33.7%), but the difference is not significant (*p* = 0.070). The proportion of satisfaction for subdomain distress relief (39.6%), rapport (32.7%) and interaction outcome (24.4) were higher among patients attended FDC clinics, but only subdomain distress relief and interaction outcome show a significant difference (*p* = 0.028 and *p* = 0.033 respectively) (Table 3).

### 3.2. Glycaemic Control among T2DM Patients

The proportion of good glycaemic control was 16.3%. Patients attended FDC clinics shows a higher proportion of good glycaemic control (19.1%) as compared to patients attended non-FDC clinics (12.7%), and the difference was statistically significant (*p* = 0.017) (Table 3).

### 3.3. Association between FDC-Implemented Clinic and Doctor-Patient Interaction Satisfaction towards Glycaemic Control among T2DM Patients in Kelantan

All the variables were explored using simple logistic regression to identify significant factors associated with glycaemic control (Table 4). The variables age, sex, race and duration of diabetes were found significantly associated with good glycaemic control. All the significant variables were then adjusted in the multivariable logistic regression models to determine the association between FDC-implemented clinic and doctor-patient interaction satisfaction towards glycaemic control. The multivariable logistic regression models were obtained by using the enter method (Table 5).

The FDC clinics were associated with good glycaemic control (Adj. OR 1.63; 95% CI: 1.08, 2.47; *p* = 0.021) when adjusted for age, sex, race and duration of diabetes. The model was fit with Hosmer Lemeshow test, *p*-Value = 0.170, classification Table 84.1% correctly classified and area under Receiver Operating Characteristics (ROC) curve was 68.5%.

There was a significant association between the satisfaction of doctor-patient interaction (overall SKIP-11) and good glycaemic control (Adj. OR 1.77; 95% CI: 1.19, 2.63; *p* = 0.005) when adjusted for age, sex, race, duration of diabetes. The model was fit with Hosmer Lemeshow test, *p*-Value = 0.078, classification Table 84.3% correctly classified and area under Receiver Operating Characteristics (ROC) curve was 68.8%. No interaction between clinic type and overall SKIP-11 (*p* = 0.107), and there were no significant interaction between all other variables.

## 4. Discussion

This study shows that satisfaction on doctor-patient interaction as well as the implementation of FDC were the important factors associated with T2DM patient’s glycaemic control. The proportion of good glycaemic control among T2DM in Kelantan was still low as compared to another local study [12] and other countries such as Mexico, Brazil and Saudi Arabia [4,16,17]. However, the comparison must be made cautiously as other study used different cut-off point such as HbA1c of <7% to be defined as good glycaemic control as compared to this study which uses cut-off point ≤6.5%. This study found that the proportion of good glycaemic control was higher among FDC clinics attendees as compared to non-FDC attendees. A local study by Jaafar et al. reported initial findings from a pilot implementation of FDC in Malaysia showed an improvement in the proportion of good glycaemic control in two selected health clinics from 31.0% to 41.7% and 7.6% to 22.7% respectively [6]. It shows that the implementation of FDC in Kelantan does have some effects towards improvement glycaemic control even though the causality cannot be proven yet. Furthermore, this study found that T2DM patients who attended FDC clinics have higher odds to achieve good glycaemic control as compared to those attended non-FDC clinics. In FDC clinics, patients will be attended by a similar set of doctors and healthcare team members according to their appointed zone [6]. Apart from that, patients were highly accessible for treatment from Family Medicine Specialist at FDC clinics through regular follow-up. This would further enhance the diabetic care, increase patients satisfaction and improve patient’s health outcome [18]. In addition, the implementation of FDC in Malaysia has enable the health status of members in a population to be captured through population survey. Following that, prevention and intervention program can be tailored according to the health needs at the community level without financial cost added to the patients [6]. The community health campaign were conducted by the same PHCT, which includes weight reduction program, quit smoking, healthy diet. Subsequently, this could result in a close relationship between the patient and the local healthcare providers as management were not only prescribed at health clinic, but also engaged at community level. Good rapport can be enhanced when the similar doctor and team attending the same patients, and thus can improve patient’s satisfaction and further increase patient’s compliance to prescribed treatment [8]. A good adherence to prescribed treatment will lead to an improvement of the clinical outcome [7,19,20]. Furthermore, if the patients were treated by doctors who had a speciality in diabetes, better quality of care in terms of process measures such as HbA1c, cholesterol level, urine analysis and blood pressure monitoring can be achieved [21]. In addition, Kelantan State Health Department has conducted yearly diabetic educator training programs since the year 2014 for selected medical assistant and nurses to function as a diabetic educator. The program has successfully added the number of diabetic-competent staffs in aiding the management for diabetic patients.

This study also observed a significant difference in SKIP-11 subdomain ‘distress relief’ and ‘interaction outcome’ between the two types of health clinics. Distress relief measures the information provided by the doctor, by which the utilization of simple language would contribute to the higher satisfaction [22]. Satisfaction on distress relief would increase patients ability and empowerment to take charge of their own diabetic control, controlling their dietary intake, perform regular exercise and compliance to medication as patients had enough information provided by the doctor and healthcare team [23]. A good interaction outcome reflects a good doctor’s communication skills and patient’s adherence intent. It will lead to patients own willingness to adhere unto pharmacological and non-pharmacological treatment, thus lead to improve glycaemic control [8,24]. The availability of resident Family Medicine Specialist could lead to higher satisfaction of distress relief and interaction outcome among FDC clinic’s attendees. However, a higher turnover rate of healthcare personnel, especially among doctors every two-yearly, pose a major challenge for FDC clinics as patients might not be seen by the same doctors throughout their management of chronic illness. Thus, the PHCT must consist of more than one doctor to avoid shortcoming [6]. Nevertheless, FDC clinics were equipped with better facilities which make patients easier and comfortable to undergone fundus examination, electrocardiogram (ECG) nor x-ray when needed, as compared to non-FDC clinics with limited facilities. The findings following those examinations can be reviewed directly by the designated PHCT, and thus proper management can be prescribed accordingly.

Apart from the above, this study observed a significant association between the satisfaction of doctor-patient interaction with good glycaemic control. The association between doctor-patient interaction and glycaemic control could be an indirect effect [25]. The satisfied patients would have good glycaemic control because they had good adherence to treatment, better engagement and attentiveness during the clinic visit, better continuity of care and the higher ability for self-care [8,9,26,27]. Providing adequate equipment for non-FDC clinics and increase the number of medical officers and diabetic educator could subsequently improve patient’s satisfaction among non-FDC attendees and thus improve their glycaemic control.

There were several known significant factors associated with glycaemic control, as repetitively described in other studies. The factors include age, sex, race, duration of diabetes, educational level and employment status [12,13,14,15,28]. In this study, factors including age, sex, race and duration of diabetes were found significantly associated with glycaemic control, thus were controlled in the analysis. The government primary care services in Malaysia is affordable, and various programs have been implemented to assist diabetic patients such as diabetic management course and medication adherence counselling. Hence, older age patients will still have the ability to access and gain benefit from these primary care services. Furthermore, the culture of having an extended family in Kelantan might contribute to care of elders’ diabetic patients, such as reminding the elders to take the prescribed medication [12]. Despite that, older people must have retired and had enough time for diabetic self-monitoring [17]. A study by Nduati et al. found that female diabetic patients had three times higher odds for poor glycaemic control as compared to male diabetic patients [15]. Meanwhile, the duration of diabetes affects the function of β cells of the pancreas and the likelihood to comply with diabetic management [28].

The strength of this study lays on the large sample size recruited from all-district in Kelantan and hence representative to Kelantan populations. This study highlighted that restructuring of primary healthcare systems into FDC-implemented health clinics had observed good glycaemic control among its diabetic attendees. However, there were has some limitations in this study by which the observed association cannot be regarded as causal inference because the study design used was a cross-sectional. Hence, the interpretation of the results must be made cautiously.

## 5. Conclusions

Higher satisfaction on doctor-patient interaction was observed among FDC clinics attendees. The satisfaction and implementation of FDC were associated with good glycaemic control among T2DM in Kelantanese population. Hence, FDC shall be strengthened, mainly in the aspect of equipment and healthcare personnel, which subsequently could improve the patient’s satisfaction and accelerate the achievement of glycaemic control.

## Figures and Tables

**Table 1:** Profile of T2DM patients attended primary health clinics in Kelantan (*n* = 772).

Variables	Total (*n* = 772)		T2DM Attended FDC Clinics (*n* = 434)		T2DM Attended non-FDC Clinics (*n* = 338)		*p*-Value
	*n*	(%)	*n*	(%)	*n*	(%)	
**Age ^a^**	60.57	(10.04)	60.41	(9.79)	60.77	(10.37)	0.609 ^b^
**Sex**							0.666 ^c^
Male	245	(31.7)	141	(32.5)	104	(30.8)	
Female	527	(68.3)	293	(67.5)	234	(69.2)	
**Race**							<0.001 ^c^
Malay	738	(95.6)	405	(93.3)	333	(98.5)	
Non-Malay	34	(4.4)	29	(6.7)	5	(1.5)	
**Marital status**							0.993 ^c^
Married	562	(72.8)	316	(72.8)	246	(72.8)	
Single/divorce	210	(27.2)	118	(27.2)	92	(27.2)	
**Employment status**							0.641 ^c^
Employed	206	(26.7)	122	(28.1)	84	(24.9)	
Unemployed	566	(73.3)	312	(71.9)	254	(75.1)	
**Duration of diabetes**							0.991 ^c^
<5 years	287	(37.2)	162	(37.3)	125	(37.0)	
5–9 years	265	(34.3)	148	(34.1)	117	(34.6)	
≥10 years	220	(28.5)	124	(28.6)	96	(28.4)	

^a^ mean(SD), ^b^ Independent *t*-test, ^c^ Chi-square statistic, FDC: Family Doctor Concept.

**Table 2:** Profile of primary health clinics in Kelantan (*n* = 20).

Variables	Total (*n* = 20)		FDC (*n* = 10)		Non-FDC (*n* = 10)		*p*-Value ^a^
	*n*	(%)	*n*	(%)	*n*	(%)	
**Average T2DM patients (per day)**							0.018
<20 patients	7	(35.0)	1	(10.0)	6	(60.0)	
20–30 patients	5	(25.0)	2	(20.0)	3	(30.0)	
≥31 patients	8	(40.0)	7	(70.0)	1	(10.0)	
**Number of medicalofficers**							0.005
One to three	6	(30.0)	0	(0.0)	6	(60.0)	
Four to six	6	(30.0)	3	(30.0)	3	(30.0)	
≥seven	8	(40.0)	7	(70.0)	1	(10.0)	
**Number of diabetic educators**							0.173
One	14	(70.0)	5	(50.0)	9	(90.0)	
Two	4	(20.0)	3	(30.0)	1	(10.0)	
≥Three	2	(10.0)	2	(20.0)	0	(0)	
**Number of pharmacists**							0.001
One to three	12	(60.0)	2	(20.0)	10	(100.0)	
Four to six	6	(30.0)	6	(60.0)	0	(0.0)	
≥seven	2	(10.0)	2	(20.0)	0	(0.0)	
**Number of dieticians**							0.474
None	18	(90.0)	8	(80.0)	10	(100.0)	
One	2	(10.0)	2	(20.0)	0	(0)	
**Number of physiotherapists**							<0.001
None	9	(45.0)	0	(0)	9	(90.0)	
One	11	(55.0)	10	(100.0)	1	(10.0)	
**Availability of fundus camera at the clinic**							0.003
No	7	(35.0)	0	(0)	7	(70.0)	
Yes	13	(65.0)	10	(100)	3	(30.0)	
**Availability of X-ray machine at the clinic**							0.005
No	11	(55.0)	2	(20.0)	9	(90.0)	
Yes	9	(45.0)	8	(80.0)	1	(10.0)	

^a^ Fisher exact statistic.

**Table 3:** Comparison of doctor-patient interaction satisfaction and glycaemic control among T2DM attended primary health clinics in Kelantan (*n* = 772).

Variables	Total (*n* = 772)		T2DM Attended FDC Clinics (*n* = 434)		T2DM Attended non-FDC Clinics (*n* = 338)		*p*-Value ^a^
	*n*	(%)	*n*	(%)	*n*	(%)	
**Overall SKIP-11**							0.070
Satisfied	288	(37.3)	174	(40.1)	114	(33.7)	
Unsatisfied	484	(62.7)	260	(59.9)	224	(66.3)	
*Subdomains SKIP-11*							
*Distress relief*							0.028
Satisfied	280	(36.3)	172	(39.6)	108	(32.0)	
Unsatisfied	492	(63.7)	262	(60.4)	230	(68.0)	
*Rapport*							0.400
Satisfied	243	(31.5)	142	(32.7)	101	(29.9)	
Unsatisfied	529	(68.5)	292	(67.3)	237	(70.1)	
*Interaction outcome*							0.033
Good	167	(21.6)	106	(24.4)	61	(18.0)	
Poor	605	(78.4)	328	(75.6)	277	(82.0)	
*Glycaemic control*							0.017
Good (HbA1c ≤ 6.5%)	126	(16.3)	83	(19.1)	43	(12.7)	
Poor (HbA1c > 6.5%)	646	(83.7)	351	(80.9)	295	(87.3)	

^a^ Chi-Square statistic.

**Table 4:** Factors associated with good glycaemic control among T2DM patients in Kelantan by simple logistic regression (*n* = 772).

Variables	Crude OR (95% CI)	*p*-Value
**Age**	1.03 (1.01, 1.06)	0.001
**Sex**		
Female	1	
Male	1.93 (1.31, 2.86)	0.001
**Race**		
Malay	1	
Non-Malay	2.23 (1.04,4.80)	0.039
**Marital status**		
Married	1	
Single/divorced	1.19 (0.78, 1.81)	0.415
**Employment status**		
Unemployed	1	
Employed	1.02 (0.66, 1.56)	0.934
**Duration of DM, years**		
<5 years	1	
5–9 years	0.69 (0.45, 1.06)	0.089
>10 years	0.36 (0.21,0.61)	<0.001
**Average T2DM patients per day**	0.99 (0.97, 1.01)	0.373

**Table 5:** Association between FDC-implemented clinic and doctor-patient interaction satisfaction towards glycaemic control by multiple logistic regression (*n* = 772).

Variables	*β*	Adjusted OR (95% CI)	*p*-Value *
**Clinic type**			0.021
Non-FDC		1	
FDC	0.490	1.63 (1.08, 2.47)	
**Overall SKIP-11**			0.005
Unsatisfied		1	
Satisfied	0.568	1.77 (1.19, 2.63)	

* Models adjusted for age, sex, race, duration of diabetes.
